# Supplementary material for: Patterns of Intron Gain and Loss in Fungi
Source: PLoS Biol. 2004 Nov 30;2(12):e422. doi: 10.1371/journal.pbio.0020422 (PMC532390; doi:10.1371/journal.pbio.0020422)
Supplement: Table S1 — Also available at http://genes.mit.edu/NielsenEtAl/. (4.3 MB ZIP). [file pbio.0020422.st001.zip › NielsenEtAl/html/651.html]

AN4763.1.NCU02015.1.MG03664.1.FG08531.1


```
 CLUSTAL W (1.82) Multiple Sequence Alignments - Introns Inserted


Sequence 1: NCU02015.1	680 aa
Sequence 2: MG03664.1	642 aa
Sequence 3: FG08531.1	679 aa
Sequence 4: AN4763.1	565 aa
Alignment Length: 719 aa
Number Identitical Residues: 197 aa
Alignment Score (without introns) 11258


MG03664.1 	------------------------------MAGPPSI--ISSRMTDINSDD-EG-NKEAQ
NCU02015.1	MASEEGPSRSASPTNVDNTFPQYPPRAELTVAGPPSL--ISSRMTDIGTEDGQGSDTTPR
FG08531.1 	---------MASKPDDDGFLAPYVSRSD--AQRPLSI--VSSRMTDIASEDGDG------
AN4763.1  	------MAFQFSNNPESNETTNNPAPHVLGLPRPPSVGGISSRVTDMSEDGDQ-------
          	       :   :    ..  .   .        * *:..:***:**:  :..:       

MG03664.1 	G----STNGNRRS--QLENL-SRPGTARTGLSSR---TQNQPLR-----HTL-GKRTSAA
NCU02015.1	GGGPSSANPNRRSGFYSDTQ-SRPETSGTGLSSRGPWAQSVPLR-----QQLTGKRGSIA
FG08531.1 	----PEVQNNRLSIPQSTDMGSRPDTARTGASSSRGPWQSQSLRNKTYLAGVQAKRGSVE
AN4763.1  	---------------------SQTNTMSSHVPHR----HSVSRR------GPPPARSSIA
          	                     *:. *  :  .      :. . *           * *  

MG03664.1 	ASS-VASGRPGSSLS---RSHVPSLTSHAFFHPMSSQKLQAQRSG-TRPTMLLRQHSRGD
NCU02015.1	GSIGSAGGRPISAAS---RSHVPSLTSHGFFRPMSSQKLQAQRGA-GRPPTMNRPY----
FG08531.1 	SSTAGSTSQPPSLSS---RSHVPSLQSHAFFRPMSSQKLQAQRGGSHRPSTMSQMSAAAS
AN4763.1  	STS-QITNRPGSSASRLSRTHIPSLAASGFFRPMSSQRLQAHRGRPATNHTVSTEDWG--
          	.:     .:* *  *  :*:*:*** : .**:*****:***:*. .     :        

MG03664.1 	IELSRDGRSVTR-------QSVTSDFTNPQMMSRLAQSQSAYDSEQ-LRPPGSRGTEATD
NCU02015.1	---VITGEAGAR-------NSVISTGS-----GRIG-AQIAEDAEL-RMP--SRGTEMTE
FG08531.1 	PSSPTSPTSPTSPRSDEHGESSSSQMRQSIISNPIAQLQRQMSNEENMRPPPSRGTEMTE
AN4763.1  	-----DQMNQNR-------RSLISNST-------FPNSLSAADQEV----PPSRGTEFTD
          	                   .*  *          : .     . *     . ***** *:

MG03664.1 	LDGGADRVTANTSPTYGHYPTGSLSESVRPLQKKPGEGRNNSLTVRVDRNYKSRDNLP-T
NCU02015.1	QET-MERMTANTSPINGFFPTSSVTDSVRPLQS-PVDARNINVEMNKDYREESPTTPPRS
FG08531.1 	QET-LDRITANTSPSHGHYPAGSLTDSVRPLQGMSSDTGHFQHSIIVDKSYKDLSNLP-S
AN4763.1  	PII-PDRIHSNASPTAN-------TTTVRSESANLIRDRERPPHLNLKVDYKGTNENE-T
          	     :*: :*:**  .       : :**. .       .    :  .   :.      :

MG03664.1 	PVRTPRSFRSSFMLPTRNGSPQTGVNRDIQGGEKLESVASTPQLSPADAVAPTANASNGE
NCU02015.1	PTRTSRSFRSSFLMPRMNESGITGSNREIEGGEKLQSAASSPNIPPQSYERQRARFKRRA
FG08531.1 	PIKTPRSFRSSFLMPGRSNDGQLSQNRSTEGAEKLSSAASSPQFRPVDSHNEQQHPRVPY
AN4763.1  	PERSPLSFLS---LQNRNAP---ADNRDSRAHARLSSADSSP----QSIEKKPELAKSRN
          	* ::. ** *   :   .     . **. ..  :*.*. *:*     .            

MG03664.1 	K--MAVAVGRNFEFF~EGNTVFFLGGRFQNTRSTPINIATGSLVVIPSILWFAFSAPWLW
NCU02015.1	KRQNRANLGKNYEYF~EGNTVFCLGGRFQNTKQRPINIATGSLIVLPCILFFIFSAPWIW
FG08531.1 	KPSQKSDLGRVHQYF~DGNTVFCLGGRWQNTRGRPINIATGIFVVVPCALFFGFEAPWLW
AN4763.1  	K-------GRNYEYF0PS------------------------------------RGPWLW
          	*       *: .::*  .                                     .**:*

MG03664.1 	ENVSPAVPITFGYLFYICISSFVHASVSDPG0ILPRNLHQFPPPDENEDPLRLGPPTTDW
NCU02015.1	HNISPAIPVTFAYLAYICVSSFLHASASDPG0ILPRNLHKFPPPEMEDSPT--GPPTTDW
FG08531.1 	NNVSPAIPIVFAYLAYICFSSFIHASVTDPG0ILPRNLHQFPPVDDDDDPLQLSPPTTDW
AN4763.1  	HNISPAIPVLFAYVFYLCFSSFIHASVVDPG0VIPRNLHQMPPVDPSQDPLAIGPPTNDW
          	.*:***:*: *.*: *:*.***:***. *** ::*****::** : .:.*   .***.**

MG03664.1 	TLIRSAESSTSAMEVPTKYCRTCNIWRPPRTHHCRLCDNCIETADHHCVWLNNCVGRRNY
NCU02015.1	VLVHSAEASTAAMEVPIKYCKTCQLWRPPRAHHCRLCDNCVETQDHHCVWLNNCVGRRNY
FG08531.1 	ALIKSAESTTAAMEVPVKHCRTCNIWRPPRAHHCRLCDNCIETHDHHCVWLNNCVGKRNY
AN4763.1  	VMVKLATSDVAAMDVPVKYCKTCSIWRPPRCYHCRVCDNCIETLDHHCVWLNNCVGRRNY
          	.::: * : .:**:** *:*:**.:***** :***:****:** ************:***

MG03664.1 	RYFFAFVTSTTLLSIYLLGSCLGQITTYASLENISVGQAIDHFRVPFALVIYGFLGFLYP
NCU02015.1	RYFFTFVSSATVLALYLIGACLAQILVYKNQHHISFGHAVNHFRVPFAMVFFGFLTFLYP
FG08531.1 	RYFFTFVTSATVLAAYLIATSLTQILLYRNRQGISFGQAVDHFRVPFALVFLGFITFLYP
AN4763.1  	RYFFAFVSTSTLLALFLLGASLAHILVYRSREGISFSDAIDKWRVPFAMVIYGALAAPYP
          	****:**:::*:*: :*:.:.* :*  * . . **...*::::*****:*: * :   **

MG03664.1 	AALMLYHVFLMARGETTREFLNSHKFLKKDRYRAFTQGNWVKNWIVVLCRPRPPTYYRFK
NCU02015.1	AALTGYHIFLMARGETTREYLNSHKFPKSDRYRAFTQANWLKNWFVVLCRPRPPTYYGFK
FG08531.1 	AALMGYHIFLMARGETTREYMNSHKFAKKERFRAFSQASVFKNFIVVLCRPRQPTYYQFK
AN4763.1  	ASLWAYHLFLVGRGETTREYLNSHKFAKADRHRPFTQGNVIRNWIAVFGRPRPPTYMQFK
          	*:*  **:**:.*******::***** * :*.*.*:*.. .:*::.*: *** ***  **

MG03664.1 	EIAVQGDQRLAARKR-SELRRAETKEGMEMQDVGPAS--VGFQGPTSR--------AAAA
NCU02015.1	VKYNQGDQRLGSHRRWQQPAVSDSKEGMEMQNVSPQLPQTGFMGPTALRNSNNGSTAEVR
FG08531.1 	AHYHEGDQRLGIRRD---KRPRSSSQGLEMHDVNPGS--SGFQGPVSLR-------NDTP
AN4763.1  	EYYQEGDQRLSTVKRRFLPRNTEPQNDIEMQHVPPPN--------SA-------------
          	    :*****.  :        ...:.:**:.* *           :             

MG03664.1 	A
NCU02015.1	A
FG08531.1 	H
AN4763.1  	-
          	
```
